# Sunitinib resistance in renal cell carcinoma

**DOI:** 10.15586/jkcvhl.2014.7

**Published:** 2014-04-22

**Authors:** Christudas Morais

**Affiliations:** Centre for Kidney Disease Research, School of Medicine, The University of Queensland at Translational Research Institute, Brisbane, Queensland 4102, Australia

## Abstract

Of the many targeted therapies introduced since 2006, sunitinib has carved its way to become the most commonly used first-line therapy for the treatment of metastatic renal cell carcinoma (RCC). Despite significant improvements in progression-free survival, 30% of the patients are intrinsically resistant to sunitinib and the remaining 70% who respond initially will eventually become resistant in 6–15 months. While the molecular mechanisms of acquired resistance to sunitinib have been unravelling at a rapid rate, the mechanisms of intrinsic resistance remain elusive. Combination therapy, sunitinib rechallenge and sequential therapy have been investigated as means to overcome resistance to sunitinib. Of these, sequential therapy appears to be the most promising strategy. This mini review summarises our emerging understanding of the molecular mechanisms, and the strategies employed to overcome sunitinib resistance.

## Introduction

The past decade has witnessed tremendous improvements in the understanding of the role of angiogenesis in renal cell carcinoma (RCC), leading to the development and implementation of many angiogenesis-inhibitors, also known as targeted therapies, in clinical practice (1–7). These achievements largely stem from the elucidation of two inter-connected molecular pathways that regulate angiogenesis and proliferation in RCC, an inactivated von Hippel Lindau (VHL) gene and activated mammalian target of rapamycin (mTOR) (6–11). These pathways have been extensively reviewed (8–18). Of the many targeted therapies, sunitinib has carved its way to become the most frequently used first-line therapy for the treatment of metastatic RCC. However, the initial enthusiasm is hampered by the development of intrinsic and extrinsic resistance to therapy. In this mini review, a summary of the angiogenesis pathway in RCC, the emerging molecular mechanisms of sunitinib resistance, and the approaches to overcome resistance to sunitinib in RCC are discussed.

## The VHL-HIF axis in RCC

**Figure 1.**
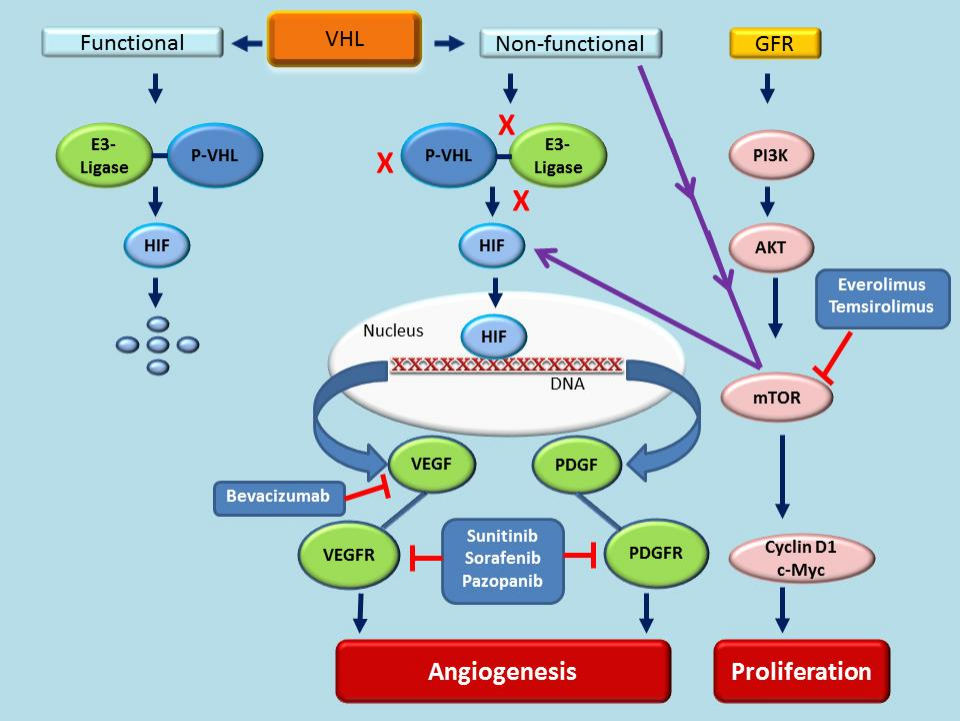
**The role of VHL and mTOR in angiogenesis and proliferation of RCC.** A non-functional VHL is the major risk factor for the development and progression of RCC. The functional protein of VHL, pVHL, complexes with E3-ligase and degrades HIF. When the VHL is non-functional, HIF is stabilized and translocated to nucleus where it binds with HIF responsive elements of the DNA and activates many pro-angiogenic factors including VEGF and PDGF. They interact with their respective tyrosine kinase receptors VEGFR (mostly at endothelial cells) and PDGFR (mostly at vascular smooth muscle cells and pericytes) and promote angiogenesis. The PI3K/AKT/mTOR pathway is activated by many factors including growth factor receptors. mTOR in turn activates cyclin D1 and cMyc and promotes cell proliferation and survival. Furthermore, VHL inactivation also activates mTOR, which in turn up-regulates HIF and subsequent angiogenesis. GFR, growth factor receptor; HIF, hypoxia-inducible factor; PDGF platelet-derived growth factor; PDGFR, receptor for PDGF; VEGF, vascular endothelial growth factor; VEGFR, receptor for VEGF; VHL,von Hippel Lindau gene.

The VHL gene is inactivated in 70–80% of sporadic clear cell RCC either through mutations, hyper-methylations or loss of heterozygosity (8–10). Subsequently, the production of its functional protein, pVHL, is either inhibited or decreased in these cases. The best studied function of pVHL is the degradation of the transcription factor hypoxia-inducible factor (HIF). As the microenvironment of solid tumors is often hypoxic, tumor cells undergo adaptive changes to facilitate their survival. One such survival mechanism under hypoxic conditions is the up-regulation of HIF-alpha (HIF-α). Under normoxic conditions, pVHL forms complexes with elongin B, elongin C, Rbx1 and cullin 2 to form a pVHL- E3 ubiquitin ligase complex (pVHL-E3 complex) (15–23). The pVHL-E3 complex then binds to HIF-α, leading to its polyubiquitination and proteasomal degradation. In the absence of a functional pVHL, secondary to VHL mutations, the formation of the pVHL-E3 complex and its binding to HIF are inhibited and therefore, the degradation of HIF-α is prevented even in normoxic conditions (15–23). This leads to the stabilization and accumulation of HIF in cells (Figure 1). Subsequently, HIF is translocated to the nucleus, where it binds to hypoxia-responsive elements of the DNA and transactivates a plethora of molecules that regulate angiogenesis (Figure 1). The best studied of these molecules is the vascular endothelial growth factor (VEGF), a crucial regulator of vascular development during embryogenesis (vasculogenesis) and blood vessel formation from the existing endothelium in adults (angiogenesis) (24–31).

## The role of VEGF in RCC

In humans, the VEGF system consists of five secreted ligands, VEGF A-D and placenta growth factor-1 (PlGF), and three receptor tyrosine kinases, VEGF R1-R3. The binding of the ligands to the receptors initiates VEGF-mediated angiogenesis which involves endothelial cell proliferation, migration, permeability and capillary formation. However, VEGF alone is not sufficient for the maintenance and stabilization of the newly formed vessels, and requires input from the surrounding microenvironment. This support comes from surrounding peri-endothelial cells such as vascular smooth muscles (VSMC) and pericytes that stabilize the newly formed vasculature and support endothelial cell survival (32–34). This is achieved by the cross talk between platelet-derived growth factor-B (PDGF-B) secreted by the endothelial cells and the receptor tyrosine kinases of PDGF, especially PDGFR-B, of the VSMC and pericytes (32–35). Thus the interplay between VEGF, PDGF and their tyrosine kinase receptors plays a crucial role in angiogenesis secondary to VHL inactivation in RCC.

## The role of mTOR in RCC

mTOR, which exists as mTORC1 and mTORC2 complexes, is a key component of the phosphoinositide 3-kinase (PI3K)/Akt signalling pathway that regulate cell cycle, proliferation and angiogenesis (12, 36). mTOR signalling can be activated via a number of mechanisms, including overexpression of growth factor receptors, mutations in PI3K/Akt, tuberous sclerosis tumor suppressor genes TSC1/2 or phosphatase and tensin homolog (PTEN) (37–42). mTOR activates the translation of the pro-proliferative factors such as cyclin D1 and cMyc, and the angiogenic factor HIF through phosphorylation of ribosomal protein S6 kinase (S6K) and eukaryotic translation initiation factor 4E-binding protein 1 (4EBP1) (6, 43–45). The mTORC2, through protein kinase Cα, regulates cell morphology, motility, adhesion, invasion and metastasis (45). Furthermore, inactivation of VHL increases mTOR activity which in turn exacerbates the loss of VHL function leading to enhanced HIF activity (38) (Figure 1). VHL inactivation itself leads to deregulation of cyclin D1 (6, 44). Thus mTOR activation is involved in cell proliferation through cyclin D1 and c-Myc, and angiogenesis through HIF (Figure 1).

## Targeted therapy in RCC

Elucidation of these pathways had identified the potential of angiogenesis inhibition as a promising therapeutic option for metastatic RCC leading to the development and implementation of angiogenesis and mTOR inhibitors in clinical practice. These targeted therapies are broadly classified as VEGF inhibitors, multi-tyrosine kinase inhibitors and mTOR inhibitors. The most successful VEGF inhibitor is the humanized VEGF-neutralizing antibody bevacizumab, which exerts its anti-angiogenic activity by acting against the angiogenic endothelial cells surrounding the tumor, rather than the tumor *per se*, thus blocking the supply of oxygen and nutrients to the tumors (46–48). The multi-tyrosine kinase inhibitors are sunitinib, sorafenib, pazopanib and axitinib. Many more are in various phases of clinical trials. They inhibit multiple tyrosine kinase receptors and neutralize the downstream signalling pathways activated by ligand-receptor binding that leads to angiogenesis. Two of the most successful mTOR inhibitors are temsirolimus and everolimus. Both are rapamycin analogues and bind to FK506-binding protein 12 (FKBP12), which in turn binds to mTOR leading to the inhibition of the PI3K/Akt/mTOR pathway (45, 49). In addition, temsirolimus has been shown to have a direct inhibitory effect on HIF and VEGF (50). Of these targeted therapeutics, sunitinib has become the most frequently used first-line targeted therapeutic for the treatment of metastatic RCC.

## Sunitinib in RCC

Sunitinib malate (SUTENT®) is a small molecule multi-tyrosine kinase inhibitor that inhibits VEGFR-1, -2 and -3, PDGFR-α and -β, stem cell factor receptor (KIT), Fms-like tyrosine kinase 3, colony stimulating factor type 1 receptor and the glial cell-line-derived neurotrophic factor receptor (RET) (51). Phase II trials of sunitinib in cytokine-refractory RCC patients showed response rates of 39–40%, stable disease rates of 23–27%, median time to tumor progression of 8.7 months, and a median survival of 16.4 months (52–55), leading to its accelerated approval for RCC in 2006. This was followed by regular approval in 2007 based on a phase III clinical trial that showed superior outcome as a first-line therapy when compared with interferon alpha in patients with clear cell RCC (1, 3, 51, 55). Subsequently, immunotherapy, which used to be the mainstay of treatment for metastatic RCC, was replaced by targeted therapy, and sunitinib has become the most commonly used first-line therapy for metastatic RCC.

## Intrinsic and acquired resistance to sunitinib

Despite the benefits achieved through sunitinib in terms of progression-free survival and disease stabilization, all patients develop resistance to sunitinib and eventually relapse. While the criteria for defining resistance or response to therapy based on RECIST (response evaluation criteria in solid tumors) are debatable as recently pointed out (56, 57), and investigators implement variations, based on the available data it is reasonable to conclude that approximately 70% of patients respond to therapy initially and the remaining 30% show primary resistance (intrinsic resistance) (57–59). In the 70% of patients who show initial response, durable responses are rare, and acquired resistance (extrinsic resistance) to treatment develops in almost all of them in 6–15 months (56–63).

The mechanisms of sunitinib resistance are varied and multifactorial. Table 1 summarizes our emerging understanding of the molecular mechanisms of resistance to sunitinib. It should be noted that most of these mechanisms are based on pre-clinical studies, and therefore, their relevance in clinical settings is yet to be verified. Taken together, the functional significance of these mechanisms can be summarized under two categories: restoration of angiogenesis through the activation of VEGF-independent pathways (64–69, 71–80) and reduced bioavailability either through increased efflux or lysosomal sequestration (70, 81). Of these, activation of tyrosine kinase-independent alternate angiogenesis pathways leading to restoration of angiogenesis appears to be gaining consensus.

**Table 1.** Emerging mechanisms of resistance to sunitinib

Parameter	Mechanism of Resistance	Ref
ATX*	Endothelial ATX activates LPA signalling to promote renal tumorigenesis	(64)
Chemokines	Down-regulation of angiostatic chemokines IFN-γ, IFN-γR and CXCL9 restores angiogenesis	(65)
COX-2	Enhanced COX-2 up-regulates HIF	(66)
EMMPRIN	High EMMPRIN causes resistance via hyaluronan-mediated activation of ErbB2	(67)
HDM2/HDMX	Inhibition of p53 by HDM2 and HDMX restores angiogenesis	(68)
IL-8	Increased plasma level leads to tumor growth and vascularity	(69)
Lysosomes	Sequestration of sunitinib in lysosomes reduces bioavailability	(70)
MicroRNA	Decreased miR-141promotes angiogenesis; increased miR-942, miR-628-5p, miR-133a, and miR-484 promote angiogenesis through up-regulation of MMP-9 and VEGF	(71, 72)
MDSC	Intra-tumoral MDSC provides sustained immune suppression and angiogenesis	(73)
NGAL	Increased NGAL activates alternate pro-angiogenic signaling pathway such as Ras-GTP, Erk1/2, and STAT1α	(74)
Polymorphism	CYP3A5 rs776746; VEGFR2 rs1870377; VEGFR3 rs307826; VEGFR3 rs307821; VEGFR3 rs448012; PDGFRA rs1800812; IL-8 rs4073; PXR rs3814055; ABCB1 rs2032582; ABCB1 rs1128503	(75–78)
PRKX	Overexpression up-regulates microphthalmia-associated transcription factor (MITF)	(79)
PTEN	Inactivation of PTEN restores angiogenesis through activation of P13/Akt/mTOR	(80)
RLIP76	Active efflux of sunitinib from cells leads to reduced bioavailability	(81)
SKI	SK1activates ERK and inhibits ATP-binding cassette (ABC) drug transporter family	(82)

* ATX, autotaxin; Cox-2, cycloxygenase-2; EMMPRIN, Extracellular matrix metalloproteinase inducer; HDM2, human double minute 2; HDMX, human double minute x; IL-8, interleukin-8; LPA, lysophosphatidic acid; MDSC, myeloid derived suppressor cells; NGAL, neutrophil gelatinase-associated lipocalin; PRKX, protein kinase x-linked; PTEN, phosphatase and tensin homolog; RLIP76, ral-interacting protein 76; SKI,sphingosine kinase-1.

While the mechanisms of sunitinib-mediated alternate angiogenesis are still elusive, hypoxia is emerging as the major culprit. Sunitinib inhibits angiogenesis, largely through the inhibition of VEGF and its receptors. This helps in the stabilization or regression of the tumor in the short term. However, this also results in hypoxia. Sustained hypoxia by sunitinib ‘resets’ the tumor microenvironment and leads to the development of a VEGF/VEGFR-independent alternate angiogenic pathway through the up-regulation of angiogenic factors other than VEGF (60, 83). For example, as shown in Table 1, up-regulation of IL-8 or down-regulation of IFN-γ (65, 69), may circumvent the anti-angiogenic effects of sunitinib, and functionally compensate for the VEGF/VEGFR-mediated inhibition of angiogenesis. Apart from contributing to acquired resistance through alternate angiogenesis pathways, hypoxia could also contribute to intrinsic resistance by selecting a more malignant RCC phenotype, which may accelerate metastatic development and prone cells to insensitivity for anti-angiogenic treatment (59, 84).

## Overcoming resistance to sunitinib

The pre-clinical studies (Table 1) have demonstrated the beneficial effects of adjunct therapy, targeted at the specific molecules identified at each study, to overcome resistance. However, their clinical relevance needs to be established. In patients, sunitinib rechallenge, combination therapy and sequential therapy have been investigated to overcome sunitinib resistance.

The rationale for sunitinib rechallenge is that resistance to sunitinib is transient and that after a short treatment interruption, sensitivity to sunitinib can be restored by subsequent rechallenge. Evidence for this comes from a pre-clinical study, a retrospective study, and a case report. In the pre-clinical study, primary RCC cells isolated from patients who were resistant to sunitinib, when grown as a mouse xenograft, responded to sunitinib (85). In the retrospective study, of the 23 patients rechallenged with sunitinib, 5 patients (22%) achieved an objective partial response, and 17 patients (74%) had stable disease (86). The median progression-free survival (PFS) was 13.7 months with initial treatment, and 7.2 months with rechallenge. Patients who had an interval of more than 6 months between sunitinib rechallenge had a longer PFS than those who started the rechallenge within 6 months (median PFS, 16.5 vs 6.0 months; P=.03). No substantial new toxicity or significantly increased severity of prior toxicity was seen during rechallenge (86). A case report by Ravaud and colleagues showed that rechallenge with sunitinib resulted in a partial response for 12 months in a 60-year old patient (61). Additional prospective studies are required to establish the beneficial effect of sunitinib rechallenge.

Combination therapy aims to overcome the limitations of a single drug either by enhancing its effectiveness on a single pathway or acting on a different molecule thereby by blocking multiple pathways (60, 87, 88). Medioni and colleagues (89) investigated a combination of sunitinib (25–50 mg) with bevacizumab (10 mg/kg), as a salvage therapy after disease progression under sunitinib monotherapy. Of the 7 patients, 2 patients showed a partial response, four had stable disease, and one patient had disease progression. The median PFS and overall survival were 8.5 and 15.1 months respectively with a tolerable toxicity profile (89). Many phase I studies have investigated a combination of sunitinib with bevacizumab (90), interferon (91), temsirolimus (92), and everolimus (60, 93). These studies were abandoned because of high degree of toxicities without any apparent benefits.

The rationale for sequential therapy is that established resistance to sunitinib may be reversed with an alternate agent that either targets the same signalling pathway, or a different pathway (56, 59, 61, 94, 95). Sequential therapies that have been tested so far include axitinib (96), pazopanib (97, 98), everolimus and temsirolimus (99). As recently summarized by Sun et al., the optimal sequential therapy appears to be sunitinib-axitinib-everolimus (100). The most promising finding is a recent case report by Raja, which employed six lines of sequential therapy in a 45-year old male patient: Sunitinib-everolimus-sorafenib-bevacizumab/vinblastine/mitomycinC-temsirolimus-bevacizumab/everolimus. This resulted in a survival of 49 months (101).

## Conclusion

Intrinsic and extrinsic resistance to sunitinib remains a challenge in the effective treatment of metastatic RCC. The mechanisms of intrinsic resistance remain elusive, and much research is warranted in this largely unexplored area. At the same time, pre-clinical studies have been unravelling the molecular mechanisms of extrinsic or acquired resistance at a rapid rate. However, there is a wide gap between the bench and the bedside. While optimism remains that this gap will narrow in the years to come, at present, the best strategy to overcome resistance to sunitinib appears to be sequential therapy. There is no universal consensus as to the optimal sequence of therapy. While establishing a universal consensus for sequential therapy appears logical, the practicality of implementing such approach would be difficult, at least in part, due the heterogeneity of RCC. Thus personalised medicine could be one way to overcome resistance. To achieve this, discovery of a sunitinib resistance ‘biomarker map’ would be of utmost value. A close interaction between clinicians and basic scientists aimed at designing clinically relevant experiments will enable a speedy resolution to overcome sunitinib resistance in the days to come.
